# “We’re sinking”: a qualitative interview-based study on stakeholder perceptions of structural and process limitations to the Canadian healthcare system

**DOI:** 10.1186/s13690-024-01279-4

**Published:** 2024-04-25

**Authors:** Jeanna Parsons Leigh, Stephana Julia Moss, Sara J. Mizen, Cynthia Sriskandarajah, Emily A. FitzGerald, Amity E. Quinn, Fiona Clement, Brenlea Farkas, Alexandra Dodds, Melanie Columbus, Henry T. Stelfox

**Affiliations:** 1 School of Health Administration, Faculty of Health, Dalhousie University, Halifax, NS Canada; 2 Department of Medicine, Cumming School of Medicine, University of Calgary, Calgary, Canada; 3 Department of Community Health Sciences, Cumming School of Medicine, University of Calgary, Calgary, Canada; 4 O’Brien Institute of Public Health, Cumming School of Medicine, University of Calgary, Calgary, AB Canada

**Keywords:** Healthcare, System, Reform, Interviews, Canada, Access, Quality

## Abstract

**Background:**

Despite longstanding efforts and calls for reform, Canada’s incremental approach to healthcare changes has left the country lagging behind other OECD nations. Reform to the Canadian healthcare system is essential to develop a higher performing system. This study sought to gain a deeper understanding of the views of Canadian stakeholders on structural and process deficiencies and strategies to improve the Canadian healthcare system substantially and meaningfully.

**Methods:**

We conducted individual, ~ 45-minute, semi-structured virtual interviews from May 2022 to August 2022. Using existing contacts and snowball sampling, we targeted one man and one woman from five regions in Canada across four stakeholder groups: (1) public citizens; (2) healthcare leaders; (3) academics; and (4) political decision makers. Interviews centered on participants’ perceptions of the state of the current healthcare system, including areas where major improvements are required, and strategies to achieve suggested enhancements; Donabedian’s Model (i.e., structure, process, outcomes) was the guiding conceptual framework. Interviews were audio-recorded, transcribed verbatim, and de-identified, and inductive thematic analysis was performed independently and in duplicate according to published methods.

**Results:**

The data from 31 interviews with 13 (41.9%) public citizens, 10 (32.3%) healthcare leaders, 4 (12.9%) academics, and 4 (12.9%) political decision makers resulted in three themes related to the structure of the healthcare system (1. system reactivity; 2. linkage with the Canadian identity; and 3. political and funding structures), three themes related to healthcare processes (1. staffing shortages; 2. inefficient care; and 3. inconsistent care), and three strategies to improve short- and long-term population health outcomes (1. delineating roles and revising incentives; 2. enhanced health literacy; 3. interdisciplinary and patient-centred care).

**Conclusion:**

Canadians in our sample identified important structural and process limitations to the Canadian healthcare system. Meaningful reforms are needed and will require addressing the link between the Canadian identity and our healthcare system to facilitate effective development and implementation of strategies to improve population health outcomes.

**Supplementary Information:**

The online version contains supplementary material available at 10.1186/s13690-024-01279-4.

**Table:** 

Text box 1. Contributions to the literature
• Despite longstanding efforts and calls for reform, Canada’s incremental approach to healthcare changes has left the country lagging behind other OECD nations.
• Canadians are aware of healthcare system distress, and desire a clear, feasible, accessible, and adaptable system.
• Stakeholder-suggested strategies included delineating roles and revising incentives, public health literacy campaigns, and interdisciplinary and patient-centred care.
• The Canadian identity is entwined with our healthcare system must be better understood to facilitate effective approaches to enhance broad health literacy to progress health reform.

## Introduction

Health experts have long anticipated a Canadian healthcare “crisis,” [1–3] citing concerns that include widespread staff shortages [4–6], long wait-times for emergency rooms [7, 8] and surgical procedures [9], as well as inconsistent and inaccessible rural care [10, 11]. Inefficiencies in healthcare systems globally have been exacerbated during the COVID-19 pandemic [7, 8, 12, 13]. High levels of post-pandemic healthcare staff burnout [14, 15], increased surgery backlogs [16], and challenges accessing care (e.g., chronic disease assessments, cancer screenings) [17] have left many Canadians concerned about the future of their healthcare system [18–20].

The Canadian healthcare system has been described as “frozen in time” as it is has been difficult to enact any widespread, substantial healthcare reform due to historical policy changes [21, 22]. Healthcare has been the responsibility of Provincial governments since the Constitution Act of 1867, harnessing advantages (i.e., increased provincial autonomy) and succumbing to disadvantages (i.e., lack of consistency in care across provinces) [21, 22]. While Canada prides itself on a healthcare system based on need, rather than ability to pay, the lack of substantial federal reform renders the country unfavourable in comparison to other OECD nations [22, 23]. Compared to other countries in the Organisation for Economic Co-operation and Development (OECD) with universal healthcare, in 2020 Canada ranked the second highest country in health spending as a percentage of gross domestic product (GDP, 12.9%), but in the middle on most measures of care (e.g., avoidable mortality) and health (e.g., life expectancy) [23, 24]. Reform to the Canadian healthcare system is essential to develop a high performing system [24, 25].

Large, nationally representative surveys have identified that most adult Canadians agree that there is need to improve the healthcare system [26, 27]. A wide range of efforts have been undertaken by governments and polling companies to consult experts and gauge public opinion to address ongoing challenges with the Canadian healthcare system [28–30]. However, many of these efforts tend to be grouped thematically, often consulting the public and healthcare experts individually to address specific problems rather than focusing on the healthcare system at large. Successful healthcare reforms often occur when patients and providers are engaged in the policy process together, with many calls for further research that includes patient perspectives [31, 32]. Thus, the main objective of this study was to develop a deeper understanding of the views of Canadian stakeholders collectively, on structural and process deficiencies and strategies to substantially and meaningfully improve the Canadian healthcare system. Interviewing stakeholders researching, making decision on, or leading healthcare reforms, in conjunction with public citizens across broad geographical regions, allowed us to harness both professional expertise and personal experiences to identify creative, multi-faceted approaches to enhance population health outcomes.

## Methods

### Study design

We applied a qualitative description design and conducted 1–1 virtual, semi-structured interviews from May 01 to August 11, 2022. The data was analyzed using inductive thematic analysis to closely examine, identify, and interpret repeating patterns of meaning [33]. Donabedian’s Model for evaluating the quality of healthcare (i.e., structure, process, outcome) was the guiding conceptual framework [34]. We conducted and reported this study according to the Consolidated Criteria for Reporting Qualitative Research checklist (Additional File 1) [35]. The University of Calgary Conjoint Health Research Ethics Board (Ethics ID#: 22–0283) and Dalhousie University Research Ethics Board (Ethics ID#: 2022–6100) approved this study.

### Participants

Using existing contacts from our professional networks, social media recruitment, and contact information available on professional websites, we targeted racially and socio-economically diverse men and women (one each) from five regions in Canada. Regions were based on federal descriptions [36], as follows: (*Atlantic Provinces*: New Brunswick, Newfoundland, Nova Scotia, Prince Edward Island; *Central Canada*: Quebec and Ontario; *Prairies*: Manitoba, Saskatchewan, Alberta; *West Coast*: British Columbia; and *Northern Territories*: Nunavut, Northwest Territories, Yukon) across four, broadly defined stakeholder groups: (1) public citizens; (2) healthcare leaders; (3) academics; and (4) political decision makers. Participants were eligible if they were English- or French-speaking adults (≥18 years) residing in Canada and were able to provide informed consent. We conducted snowball sampling from individuals who agreed to participate in the study and recruitment targets were 10–12 participants per stakeholder group or 6-months after commencing recruitment, whichever came first [37]. All individuals who agreed to participate were compensated with a $20 e-gift card.

### Data collection

A 45-minute semi-structured interview guide was developed iteratively by a professionally diverse research team that included six academics and researchers (JPL, SJMo, EAF, AQ, RD, BF) and two health leaders (FC, HTS). The development of the guide was informed by existing literature on the topic of healthcare reform among OECD nations. It was pilot tested with two health leaders (NJ, VO) and two public citizens (KM, MC) to ensure clarity of interview questions and relevance to the study objective (Additional File 2). Participants who pilot tested the interview guide received a $20 e-gift card for their time. Pilot testing resulted in minor refinement to improve language and conversational flow.

Two female research assistants (SJMi, MS), trained in qualitative methods, conducted interviews via Microsoft Teams (without video) and recorded audio with the Teams integrated audio platform. The two research assistants introduced themselves within their professional role and their institutional affiliation. The mean interview time was 33.7 min (standard deviation 11.9 min) and digitally recorded audio files were produced into verbatim transcripts via a transcription company (www.Rev.com). The textual data were reviewed, cleaned, and deidentified (SJMi, CS, AD, MS) before analysis.

### Data analysis

Transcripts were analyzed using inductive thematic analysis and managed through NVIVO 12 (QSR International). Two researchers (SJMi, CS) reviewed and coded a small sample of five transcripts independently and in duplicate using open coding [38]. Weekly progress meetings were held with a senior qualitative researcher (SJMo) wherein initial codes and a draft codebook was discussed. Two researchers (SJMi, CS) analyzed an additional five transcripts using both open and axial coding [38], iteratively refining the codebook until all relevant ideas were included. A meeting was held after completing the first round of coding for all transcripts (SJMo, EAF, AQ, FC, BF, HTS) to address new codes, consolidate ideas, and rectify disagreements by consensus. Drawing on the combined insights of those “handling” the data closely (SJMi, CS) combined with the expertise of senior members of the research team (JPL, SJMo, FC, HTS), provided a wider perspective of methodological, health policy, and healthcare systems issues.

The complete data set was then coded in duplicate (SJMi, CS) with the finalized codebook. The final round of coding informed the creation of themes which were then mapped to the Donabedian model [34]. The careful use of memos (by SJMi, CS) during initial stage of analysis provided a visible “audit trail,” moving from “raw” data, through interpretation, to the production of findings. Data analysis proceeded past data saturation that was defined as a cease in the development or expansion of already identified codes or themes (i.e., the emergency of data redundancy) [39]. Data from the four pilot interviews were not included in the final data set for analysis due to the level of content changes that were made to the guide during refinement.

## Results

**Fig. 1:**
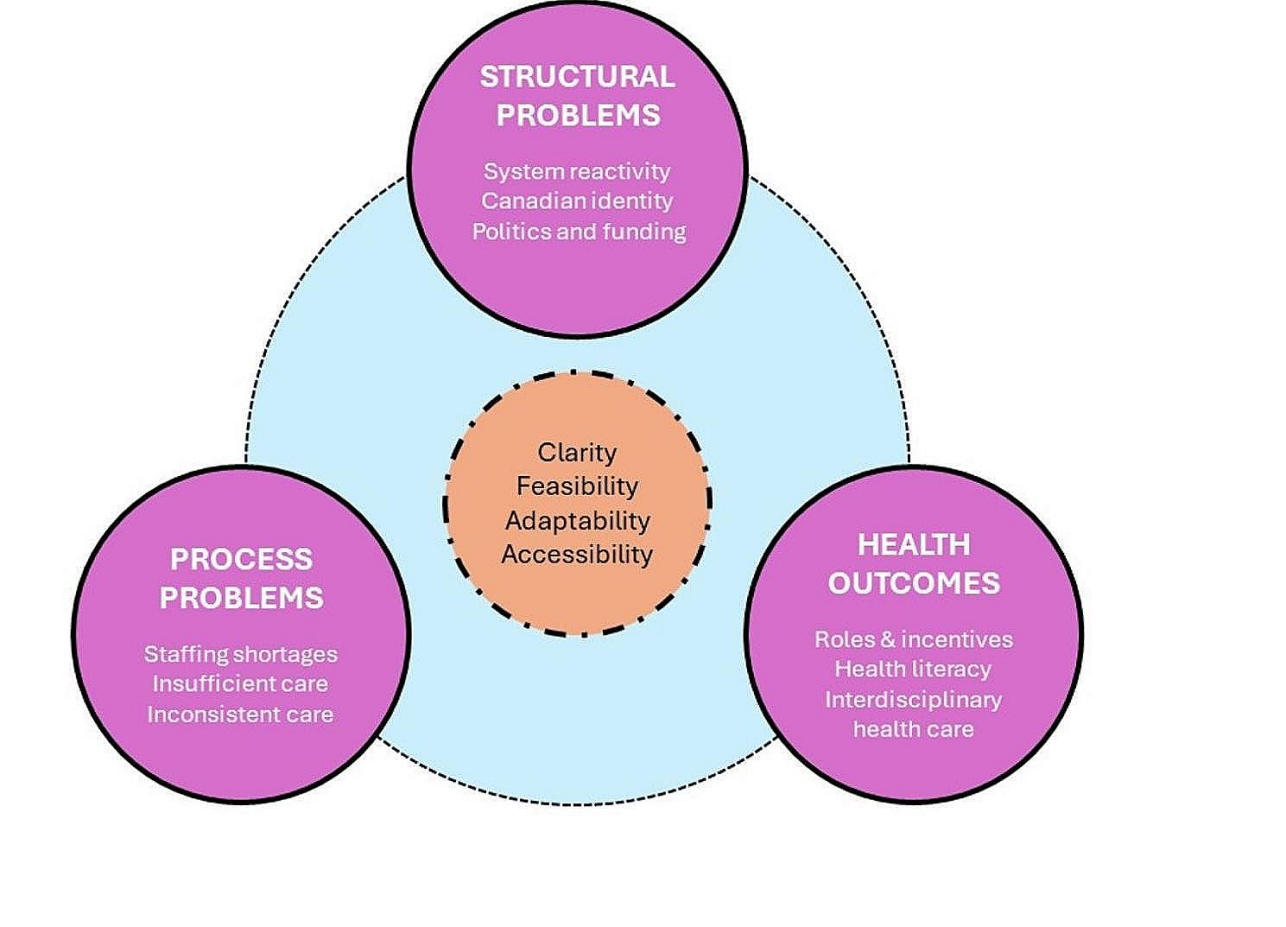
Perspectives from Canadian stakeholders on structural and process problems, opportunities to improve health outcomes, and key influential factors that are grounded conceptually in the Donabedian Model

Of 31 participants interviewed, 13 (41.9%) were public citizens, 10 (32.3%) were healthcare leaders, 4 (12.9%) were academics, and 4 (12.9%) were political decision makers. Seventeen (54.8%) participants identified as female (sex) and seventeen (54.8%) participants identified as women (gender). Twenty-four participants (80.0%) identified as white and 19 (65.5%) were aged 25–64 years (Table 1). Participants were most frequently from central (i.e., Ontario, Quebec) Canada (10, 32.3%) and prairie (i.e., Alberta, Saskatchewan, Manitoba) provinces (10, 32.3%). Nearly half of participants (13, 43.3%) had a post-graduate degree, and 20 (64.5%) participants were employed full-time, all of whom (20, 100%) were entitled to healthcare benefits through their employer, or a disability or retirement plan. Participants in our sample identified three overarching themes related to the structure of the healthcare system (1. system reactivity; 2. linkage with the Canadian identity; 3. political and funding structures), three themes related to healthcare processes (1. staffing shortages; 2. inefficient care; 3. inconsistent care), and three strategies to improve short- and long-term population health outcomes (1. delineating roles and revising incentives; 2. enhanced health literacy; 3. interdisciplinary and patient-centred care) (Fig. 1).

**Table 1:** Participant demographics and characteristics

Characteristic	Total (*N* = 31)		Academic (*n* = 4)		Decision Maker (*n* = 4)		Health Leader (*n* = 10)		Public Citizen (*n* = 13)	
	n/total	%	n/total	%	n/total	%	n/total	%	n/total	%
*Age category (years), n/total, %*										
Young adults (18-24)	4/29	14%	0/4	0%	0/4	0%	0/8	0%	4/13	31%
Adults (25-64)	19/29	66%	3/4	74%	2/4	50%	8/8	100%	6/13	46%
Seniors (65+)	6/29	21%	1/4	25%	2/4	50%	0/8	0%	3/13	23%
*Sex, n/total, %*										
Female	17/31	54%	3/4	75%	1/4	25%	5/10	50%	8/13	62%
*Education, n/total, %*										
Undergraduate	7/30	23%	1/4	25%	0/4	0%	1/10	10%	5/13	38%
Graduate	6/30	20%	1/4	25%	2/4	50%	3/10	30%	5/13	38%
Post-graduate	13/30	43%	2/4	50%	1/4	25%	5/10	50%	3/13	23%
College/trades	3/30	10%	0/4	0%	0/4	0%	0/10	0%	0/13	0%
Post-secondary	1/30	3%	0/4	0%	1/4	25%	0/10	0%	0/13	0%
*Province, n/total, %* ^*a*^										
West coast	5/31	16%	1/4	25%	1/4	25%	0/10	0%	3/13	23%
Prairies	10/31	32%	0/4	0%	2/4	50%	4/10	40%	4/13	31%
Central	10/31	32%	2/4	50%	0/4	0%	3/10	30%	5/13	38%
Atlantic	5/31	16%	1/4	25%	1/4	25%	2/10	20%	1/13	8%
Territories	1/31	3%	0/4	0%	0/4	0%	1/10	10%	0/13	0%
*Ethnic background, n/total, %*										
White	24/30	80%	4/4	100%	3/4	75%	7/9	78%	10/13	77%
Asian	3/30	10%	0/4	0%	1/4	25%	1/9	11%	1/13	8%
Indigenous	2/30	7%	0/4	0%	0/4	0%	1/9	11%	1/13	8%
*Employment Status, n/total, %*										
Full-time	24/31	65%	3/4	75%	2/4	50%	10/10	100%	5/13	38%
Part-time	2/31	7%	1/4	25%	0/4	0%	0/10	0%	1/13	8%
Retired	6/31	19%	0/4	0%	2/4	50%	0/10	0%	4/13	31%
Disability	3/31	10%	0/4	0%	0/4	0%	0/10	0%	3/13	23%
*Employer-Sponsored Extended Healthcare benefits, n/total, %*										
Yes	20/30	67%	3/4	75%	3/4	75%	8/10	80%	6/12	50%

^a^Atlantic Provinces: New Brunswick, Newfoundland, Nova Scotia, Prince Edward Island; Central Canada: Quebec and Ontario; Prairies: Manitoba, Saskatchewan, Alberta; West Coast: British Columbia; and Northern Territories: Nunavut, Northwest Territories, Yukon

Denominators that do not equal the stratified sample sizes are due to missing data

### Structural problems

#### System reactivity

Nearly all participants across the four stakeholder groups perceived the current Canadian healthcare system to be a “sick care” system. That is, the system waits for an individual to become sick before it kicks into reactive action; participants commented on how, for the most part, the system was not initially designed to help prevent the onset of disease but instead to diagnose and treat illness. As one citizen commented:

“*We call it a health system, but it’s really a caring for the sick people system and there’s not a lot of focus on health promotion and prevention as part of that system.”– (P14) Public Citizen, Female, Central Canada*.

Many participants described experiences with increased wait times for emergency rooms and ever-extending surgical waitlists—augmented by the COVID-19 pandemic—and commented on the systemic financial burden of treating severely ill patients. Many participants advocated that the best solution to the growing crisis of chronic disease and the aging population was a model focused on preventing the onset of illness. This model would involve shifting healthcare resources upstream to strengthen the existing public health infrastructure. As one healthcare leader explained:

*“We do not have an upstream approach to build in systems of support for [patients]. We’re often in a situation where we’re responding or bringing in health support, health service support, when they’re in their most acute or severe point of need. Which is quite costly and it also requires complex treatment.”– (P17) Healthcare Leader, Female, Central Canada*.

#### Linkage with the Canadian identity

Many participants reflected on the healthcare system as engrained within the Canadian identity and expressed feelings of discomfort regarding privatization of healthcare; some participants predicted a widened gap in access based on individual class and privilege:

*“What I’m worried about is that these services will become more and more privatized and not accessible. So those of us who have financial and health barriers, like we can’t physically get to the appointments, we have depression or anxiety, or we don’t have money to take the cab to get [there]”– (P24) Citizen, Female, Central Canada*.

There was a sense that Canadians had a duty to “protect” the public model as it was something that “defines us in some way” *(P1, Male, Central Canada).* Healthcare leaders described how this identity reduces the likelihood of politicians pledging substantial changes, due to perceived protectiveness from constituents:

*“It’s almost impossible to challenge whenever anybody politically says something, they’re immediately accused of creating a two-tier system”– (P20) Healthcare Leader, Male, Atlantic Provinces*.

When asked to compare the Canadian healthcare system internationally, many participants looked south to the United States. However, as one leader explained, comparing the Canadian healthcare system to that of the United States resulted in an inaccurate perception of systemic effectiveness, providing a false sense of security:

*“I think people tolerate a fairly shabby system in Canada. Right? And so we often are like, “We don’t want to be… Look at the US, it’s worse.” I’m like, “Yes, U.S is worse. I agree.” But it makes it hard to adopt things.”– (P13) Healthcare Leader, Male, Prairie Provinces*.

Among stakeholder groups, public citizens most frequently mentioned the United States, while only a minority of academics referred to the American system.

#### Political and funding structure

Healthcare funding was discussed frequently by participants. Whereas some participants acknowledged Canada’s high healthcare spending, others perceived lack of funding in areas such as preventative care, allied health services, and mental health services:

*“I don’t believe that the system is broken down, I believe that the funding and support….has become reduced, and reduced, and reduced.”- (P24) Public Citizen, female, Central Canada*.

A desire for improved funding was mentioned by the majority of public citizens while none of the decision makers interviewed mentioned wanting to improve funding. Participants discussed the costs and benefits of private and public healthcare funding; healthcare leaders were the stakeholder group that most frequently voiced concerns about privatization, particularly in relation to how publicly unpopular the concept is as it appears to contradict prevailing national values. Interestingly, a minority of public citizens interviewed voiced general concern with privatization. While only one public citizen supported privatization completely, many acknowledged that Canada’s current system is mixed while describing possible advantages to accessing more private services.

*“I don’t know, the Canadian in me is like, ‘Oh, we can’t have two tier health,’ but we kind of do already in terms of labs and those types of things. So, some way to preserve the universality of access to acute care… but for those of us that can afford to access a certain suite of services and pay for them either through our plans or through out of our pocket, to allow us to do that still within the same system”– (P14) Public Citizen, Female, Central Provinces.*

Some discussed how privately funded care might lighten the load on healthcare services, while others feared it would threaten equity and accessibility of care. Participants also regarded the organizational structure of the Canada Health Act as a barrier to providing health care that is responsive to regional needs while being consistent nationally. The current Canadian healthcare system was described as “fragmented” and a “non-system” (*P14, Female, Central Canada*), an issue that many participants felt was exacerbated by the lack of a centralized medical information system. One public citizen shared her experience of being “cut-off from follow-up” when receiving care across several provinces (*P10, Female, West Coast*). Further, participants perceived that the politicization of healthcare may encourage politicians to adopt policies based on popularity rather than scientific evidence. Many participants felt that the four-year political cycle was a significant barrier to long-term change. One healthcare leader commented on political decision making during the COVID-19 pandemic:

*“It’s not effective or helpful when governments come in and are regularly turning over leadership of the healthcare systems. If you look at what they did in Alberta… Which I think was completely stupid. You can quote me on that. They let [her] go, as their CEO. This was a person who stabilized the Alberta healthcare system, has moved that system so far ahead…The reason she was…let go, was because of COVID and the politicization of COVID.”– (P21) Healthcare Leader, Male, Prairie Provinces*.

### Process problems

#### Staffing shortages

The majority of Canadians in our sample referred to staffing shortages as a pressing and persistent problem that impacts care delivery. These workforce shortages were recognized and felt across the broader healthcare system—from hospitals to home and community care, long-term care, and primary care—resulting in low staff-to-patient ratios and high overall workloads. Public citizens commented frequently on their challenging experiences with accessing family physicians which was associated with a retiring population and a perceived lack of appropriate incentivization for those considering practicing family medicine. Participants perceived the downstream impact to include challenges with preventative care, managing chronic health disorders, and obtaining specialist referrals. One public citizen voiced:

*“If we think about me, someone who maybe in a couple years is going to want to start having kids, you have that first conversation with your family doctor. You don’t go to the emergency room and say you want to start having kids. So it’s really problematic to me that access is so poor.”– (P30) Public Citizen, Female, Central Canada*.

#### Inefficient care

Many participants described concerns regarding efficiency of the healthcare system, in particular the duration of time often required to see a healthcare professional. As one healthcare leader described:

*“I think we have access, i.e., the door is open. I think once the door is open, the time that we spend waiting for care to start, progress and end can be considerable.”– (P19) Healthcare Leader, Female, Prairie Provinces*.

For many participants, this delay resulted in feelings of anxiety and mistrust of the healthcare system. Participants who perceived inefficiencies due to duplication of services or lack of clarity regarding scope of practice cited various experiences such as redoing bloodwork, redundant consultations, and tasking healthcare professionals with paperwork. Communication challenges were described as resulting in duplication of services and increased wait times for referrals. A lack of centralized information systems was perceived to be a cause of these challenges, thereby impeding communication between healthcare professionals placing onus on patients themselves. Many participants working within the healthcare system expressed exasperation with this gap in technology and indicated that Canada lags behind OECD counterparts.

#### Inconsistent care

A common sentiment across all participant groups regarded inconsistencies in financial coverage and the type of care offered across regions; tertiary services (e.g., dental, prescription drugs) were often considered financially unattainable. Coverage across provincial health authorities, especially for chronic illnesses, was a common point of confusion. One public citizen described challenges he faced while managing diabetes:

*“Diabetic supplies… (are) all covered by Saskatchewan healthcare. The testing and the blood glucose stuff, that’s all covered, and the testing supplies. If you come to Alberta, it’s not covered. People that live near the border drive from Alberta to Saskatchewan and get all their supplies there. Why is it that Canadians that live less than a hundred kilometers apart are treated drastically different?”– (P2) Public Citizen, Male, Prairie Provinces*.

Additionally, concerns were raised about inconsistent care as it related to race and ethnicity. Some participants commented on racism and prejudice in the healthcare system, particularly towards Indigenous peoples. One healthcare leader described how a lack of Equity, Diversity, and Inclusion (EDI) in the healthcare system may contribute to erosion of trust among public citizens due to lack of linguistic or cultural understanding:

*“…new Canadians, don’t necessarily feel safe in [this] environment, because of not understanding the linguistic or cultural understanding of their own customs around their way of life and traditions. That’s a big problem, when the Canadian healthcare system, the way it’s set up, is not allowing for that diversity to be considered in any policy development, or program service development.”– (P18) Healthcare Leader, Male, Prairie Provinces*.

### Opportunities for improved health outcomes

#### Delineating roles and revising incentives

Participants highlighted the importance of clearly delineating roles and defining the scope of practice to minimize redundancies and maximize expertise. They suggested that role delineation may assist healthcare professionals with understanding what part of the care process they are accountable for and enhancing the scope of practice for better patient-centred care.

*“So our admin staff, are we ensuring that we’re really clear about what their skillset is and what their roles and responsibilities are? And are we allowing those individuals to support the system maximally and not giving pieces of their work to a clinician who should be focused exclusively clinically?”- (P19) Healthcare leader, Female, Prairie Provinces*.

Several participants suggested revising incentivization packages as a potential approach to retention of professionals within the Canadian healthcare system. They suggested re-structuring current pay models for family physicians to account for their significant case load and the cost of overhead. Many participants described how financial anxieties can compound stress associated with practicing family medicine, potentially pushing them out to pursue a specialty perceived to be more financially sustainable. One academic recommended:

*“You need enough family doctors, and you need to have a system for compensation, for paying them in a way that actually helps them stay and settle and do the job well”– (P7) Academic, Female, Atlantic Provinces*.

#### Enhanced health literacy

Public health literacy was mentioned frequently as a defining factor for the future of the Canadian healthcare system and a significant factor in healthcare disparity and equity that must be addressed in any future healthcare reform. To support this change, it was suggested that providers must engage with and be accountable to key stakeholders, including patients and the Canadian population at large. Healthcare leaders and decisionmakers suggested that enhanced public understanding of health policies, including their short- and long-term impacts, might in turn facilitate more effective public voting power:

*“I think we do need to educate the population…. Governments rise and fall on healthcare, in Canada. As long as the healthcare system is linked to the political will and people can leverage that I think we’re going to continue to struggle with this.”– (P21) Healthcare Leader, Male, Prairie Provinces*.

#### Interdisciplinary and patient-centred care

Many participants underscored the importance of shifting our healthcare system toward one that provides preventative care. They suggested that interdisciplinary collaborations might increase the ability to accurately address a patient’s individual needs thereby resulting in improved treatment processes and better adherence to treatment. Relatedly, there was concordance among all stakeholders in term of favoured interventions to enhance patient-centred care. It was suggested that shifting power from politicians to healthcare professionals and using quality indicators to make comparisons (i.e., benchmarking) would ensure progress in establishing integrated patient-centred care and that care provided is reflective of the patient perspective. One healthcare leader described:

*“…We [need] to have a bigger vision, longer vision, and evidence-based things in medicine and even in economics…down the line 20 years from now, we’re going to show better health outcomes.”– (P1) Healthcare Leader, Male, Central Canada*.

## Discussion

We present in-depth qualitative data from a large and diverse group of Canadian stakeholders on structural and process problems and opportunities to improve the Canadian healthcare system. Findings from our study suggest that Canadians in all stakeholder groups are well aware of system distress across the country, sharing experiences with inefficient and inconsistent care in a generally reactive system as well as perspectives on staffing shortages, various political and funding structures, and the linkage between our healthcare system and the Canadian identity. When discussing ongoing issues with the healthcare system, there was consensus across stakeholder groups which underscores how these systemic and process issues impact Canadians regardless of their professional proximity to the healthcare system. Despite the varying professional backgrounds of stakeholders, and geographical regions of public citizens, that their suggestions for opportunities to improve health outcomes centered around tackling similar core challenges indicated that Canadians generally have carefully considered their current healthcare system and what could be done to improve it. That the majority of our sample was public citizen stakeholders highlights their relative readiness to engage in discussions about their healthcare system. Participants in our sample desired a healthcare system that is clear, accessible, feasible, and adaptable, suggesting multiple strategies for improvement that included delineating roles and revising incentives, campaigns to enhance public health literacy, and interdisciplinary care with interventions to provide patient-centred care. Further research is needed to better understand the alignment between our national identity and healthcare to develop, refine, and implement approaches to improve broad public health literacy. While there is no silver bullet for a crisis decades in the making [40–42], Canadians are calling for tough decisions to be made.

Extensive prior research indicates that Canada’s incremental approach to healthcare changes has left Canada lagging far behind other OECD nations, however these results are not for lack of research or calls to action [22, 30, 43]. Over the past few decades, many researchers have looked closely at healthcare reform in Canada and why it has been so difficult to enact [21, 44]. By nature, Canada’s democratic governance structure forces compromise which limits sudden, substantial change. Furthermore, its healthcare landscape is shaped by numerous factors including government officials, the media, public citizens, hospital boards, medical associations, and many others, which often have opposing values and expectations [45]. Following a decision in the 1990s by multiple provincial governments to tighten healthcare budgets, Canadians voiced growing concerns about increasing wait-times [45]. There was also widespread discussion about subsidizing prescription medications with some provinces, like Quebec, pursuing increased coverage while others, like Saskatchewan, rolled back coverage [45]. Over 20 years later these concerns have not abated as Canadians are still waiting significantly longer for appointments with healthcare specialists compared to other OECD nations while continuing to be the only high income county with universal healthcare that does not cover prescription drugs [22]. Hospital re-admissions, lack of preventative care, administrative overhead [46, 47], as well as inefficiencies and inconsistencies in service offerings and coverage across provinces [10, 12, 22], have been reported as primary inefficiencies within the Canadian healthcare system, with public opinion polls highlighting that Canadians are feeling the burden of these problems regularly [30]. Our study aligns with earlier findings suggesting that staffing shortages are a significant structural shortcoming [3, 11, 48] that have been intensified by the COVID-19 pandemic [4–6] and impact patients, healthcare professionals, and allied staff [4, 5]. Canadians in our sample also commented frequently on the lack of a pan-Canadian electronic medical record database that would make patient data more accessible and organized, helping the healthcare system to operate more efficiently and save more lives. Echoing others, the lack of a national electronic medical record database was perceived to have created a disjointed system that makes accessing data a time-consuming process that has rendered Canada’s system far outdated [47, 49]. To develop a national electronic medical record database in Canada, improved collaboration and co-operation—between all levels of government, healthcare professionals, and patients—is needed [50, 51].

Canada’s healthcare system is an unrivaled pillar of national identity with close to 90% of Canadians perceiving that eliminating it would cause a fundamental change to their identity that hinges on “universal healthcare.” [22] It has been argued that universal healthcare has become entwined with our national identity—the notion that access to healthcare should be based on need, not ability to pay—resulting in a general aversion to any substantial reform [45, 52]. This aversion highlights the ongoing challenge of democratic liberal governments who must balance the logics of political and scientific accountability [44]. The defining national value of “universal healthcare” underscores an implicit social contract between the Canadian public, healthcare professionals, and government that demands an ongoing and shared commitment to equity and solidarity [22, 53]. Our findings indicate that Canadians want to set the record straight about their healthcare system—including inefficiencies and areas requiring change. Other studies echo this, highlighting how citizens are actively engaging with public health research and interested in learning new ways to re-organize the system [30, 54]. Although coverage is portable across the country, administration and service delivery are highly decentralized; [55] the Canadian healthcare system is less a true national system than a decentralized collection of jurisdictional insurance plans covering select services that are covered at the point of care [56]. Decentralization of the governance of health systems has been implemented as a possible approach for health reform or as a preferred management strategy with the overall objective to improve health system performance [57]. It is argued that decentralization empowers regional governments, enhances the efficiency of technical resources, increases accountability, and engages local populations to tailor the healthcare system to be responsive to their needs [58]. Critics of decentralization posit that this type of reform has been in response to broader, global pressure on governments by international agencies to address their failure to deliver high quality and accessible healthcare services [58]. Nonetheless, research on decentralization has found that its impact on health-related inequities are varied and depend on the pre-existing socioeconomic and organizational contexts [59, 60]. That our data suggests conflict between tailoring healthcare to individual provinces and balancing pre-existing health inequities highlights that the impact of decentralization across Canadian provinces may equally vary. The data presented in our study also provides a fundamental base for the development of future population-based research focused on further understanding the relationship between the Canadian identity and both our current healthcare system and the social determinants of health [61]. Citizen engagement in system reform—a joint effort by governments, healthcare professionals, and the public—will be crucial to successfully achieve a high-quality, evidence-based system that is grounded in shared Canadian values.

Health literacy, the ability to access and use health information to make appropriate health decisions and maintain basic health, has emerged as an important factor in population health disparities [62, 63]. Statistics Canada determined that only 40% of adult Canadians have the required level of health literacy to maintain good health [64] while the Public Health Agency of Canada reported that 60% of adults and 88% of seniors are not health literate [65, 66]. In 2013 the World Health Organization stated that low health literacy is associated with riskier health choices, decreased participation in health-promoting and disease-detection activities, poor management of chronic diseases, and lower adherence to medication [67]. Conversely, improving health literacy has a positive effect, promoting resilience among communities and individuals and empowering the public to make preventative health decisions and better self-manage [68]. Governments and organizations in Canada can take action by using plainer language and clearer communication, avoiding medical jargon, defining medical and scientific terms, and organizing information as easy to follow. Decision makers and health leaders need to take strong leadership roles to develop and implement health literacy promotion policies with sustained funding and coordination across sectors and with regular surveillance [69]. Enhancing and assessing health literacy (both strengths and needs) is particularly important to empower vulnerable populations to engage in early and sustained health promoting actions [70]. 

We conducted in-depth, interviews with a broad range of stakeholders with backgrounds across five regions in Canada. This geographically diverse sample permitted identification of similarities in perspectives and experiences, despite demographic and geographical differences, highlighting the widespread nature of shortcomings and problems raised. However, despite seeking racially and socio-economically diverse participants our sample was predominantly white, middle-aged, and well-educated; their health literacy was not necessarily reflective of the general population. Despite repeated attempts to sample participants evenly across stakeholder groups, there was considerable variability due to differing levels of engagement in the study which limited the ability to compare across stakeholder groups. Our interview guide was developed and refined iteratively including broad content expertise in qualitative design, health services research, and health economics. Nonetheless, all interviews were conducted in English, potentially limiting perspectives of non-English speakers in Canada.

## Conclusions

Canadians in our sample were well aware of the signs of healthcare system distress across all jurisdictions. They noted structural and process of care delivery problems. A defining feature of our future will be that the Canadian identity is deeply entwined with our healthcare system; this relationship must be studied, unpacked, and better understood to facilitate effective approaches to enhance broad health literacy so that we can move forward with health reform. Encouraging further interdisciplinary collaboration is the first step to informing which actions can be taken to address the many multi-level challenges that our healthcare system has been facing for decades. Programs like the Health System Sustainability Initiative out of the O’Brien Institute of Public Health highlight the value in gathering, conducting and disseminating evidence-informed recommendations that improve the quality and sustainability of Canada’s healthcare system [71–74]. Canadians are calling for reform and we have a population ready for change—the time for action is now.

### Electronic supplementary material

Below is the link to the electronic supplementary material.

Supplementary Material 1

Supplementary Material 2

## Data Availability

The data are not publicly available due to them containing semi-identifiable information that could compromise research participant privacy. Additional summary tables of count data are available from the corresponding author upon request.

## Declarations

### Data sharing and availability of materials

The data are not publicly available due to them containing semi-identifiable information that could compromise research participant privacy. Additional summary tables of count data are available from the corresponding author upon request.
