# Supplementary material for: “We’re sinking”: a qualitative interview-based study on stakeholder perceptions of structural and process limitations to the Canadian healthcare system
Source: Arch Public Health. 2024 Apr 25;82:56. doi: 10.1186/s13690-024-01279-4 (PMC11044548; doi:10.1186/s13690-024-01279-4)
Supplement: Supplementary file 1 — Supplementary Material 1 [file 13690_2024_1279_MOESM1_ESM.docx]

**Additional File 2: COREQ Checklist**

**Consolidated criteria for reporting qualitative studies (COREQ): 32-item checklist**

Developed from:

Tong A, Sainsbury P, Craig J. Consolidated criteria for reporting qualitative research (COREQ): a 32-item checklist for interviews and focus groups. *International Journal for Quality in Health Care*. 2007. Volume 19, Number 6: pp. 349 – 357

| **No. Item** | **Guide questions/description** | **Responses** | **Reported on Page #** |
| --- | --- | --- | --- |
| **Domain 1: Research team and reﬂexivity** |  |  |  |
| *Personal Characteristics* |  |  |  |
| 1. Interviewer/facilitator | Which author/s conducted the interview or focus group? | SJMi | Page 6 (Methods) |
| 2. Credentials | What were the researcher’s credentials? E.g. PhD, MD | JPL (PhD), SJMo (PhD), SJMi (MA), CS (MPH), EAF (MSc), AQ (PhD), FC (PhD), BF (MSc), AD (MSc), MC (MSc), HTS (PhD). | Not applicable |
| 3. Occupation | What was their occupation at the time of the study? | JPL (Associate Professor), SJMo (Senior Research Associate), SJMi (Research Assistant), CS (Research Assistant), EAF (Research Associate), AQ (Post-doctoral Fellow), FC (Professor, Director), BF (Senior Research Associate), AD (Research Assistant), HTS (Professor, Director). | Not applicable |
| 4. Gender | Was the researcher male or female? | Female: JPL, SJMo, SJMi, CS, EAF, AQ, FC, BF, AD, MC  Male: HTS | Page 6 (Methods) |
| 5. Experience and training | What experience or training did the researcher have? | All researchers had experience with qualitative research methods | Page 6 (Methods) |
| *Relationship with participants* |  |  |  |
| 6. Relationship established | Was a relationship established prior to study commencement? | No | Not applicable |
| 7. Participant knowledge of the interviewer | What did the participants know about the researcher? e.g. personal goals, reasons for doing the research | Participants were informed of the goal | Additional File, Page 6 (Additional File 2) |
| 8. Interviewer characteristics | What characteristics were reported about the interviewer/facilitator? e.g. Bias, assumptions, reasons and interests in the research topic | Interviewers (SJMi, MS) were qualitatively trained with a professional interest in healthcare | Page 6 (Methods) |
| **Domain 2: study design** |  |  |  |
| *Theoretical framework* |  |  |  |
| 9. Methodological orientation and Theory | What methodological orientation was stated to underpin the study? e.g. grounded theory, discourse analysis, ethnography, phenomenology, content analysis | Thematic analysis | Page 5 (Methods) |
| *Participant selection* |  |  |  |
| 10. Sampling | How were participants selected? e.g. purposive, convenience, consecutive, snowball | Purposive and snowball sampling | Page 6 (Methods) |
| 11. Method of approach | How were participants approached? e.g. face-to-face, telephone, mail, email | SJMi and LL emailed invitations using existing contacts from our professional networks, social media recruitment, and contact information available on professional websites | Page 5 (Methods) |
| 12. Sample size | How many participants were in the study? | 31 | Page 7 (Results), Page 23 (Table 1) |
| 13. Non-participation | How many people refused to participate or dropped out? Reasons? | None | Not applicable |
| *Setting* |  |  |  |
| 14. Setting of data collection | Where was the data collected? e.g. home, clinic, workplace | Interviews were conducted over TEAMs (without video) | Page 6 (Methods) |
| 15. Presence of non-participants | Was anyone else present besides the participants and researchers? | No | Not applicable |
| 16. Description of sample | What are the important characteristics of the sample? e.g. demographic data, date | Demographic and geographic data is presented on the sample as a whole | Page 23 (Table 1) |
| *Data collection* |  |  |  |
| 17. Interview guide | Were questions, prompts, guides provided by the authors? Was it pilot tested? | The guide was pilot tested with two healthcare leaders (NJ, VO) and two public citizens (KM, MC) | Page 6 (Methods) |
| 18. Repeat interviews | Were repeat interviews carried out? If yes, how many? | No | Not applicable |
| 19. Audio/visual recording | Did the research use audio or visual recording to collect the data? | Interview audio was recorded | Page 6 (Methods) |
| 20. Field notes | Were ﬁeld notes made during and/or after the interview or focus group? | Yes | Not applicable |
| 21. Duration | What was the duration of the interviews or focus group? | The mean interview duration was 33.7 minutes (11.9 SD) | Page 6 (Methods) |
| 22. Data saturation | Was data saturation discussed? | Yes | Page 6 (Methods) |
| 23. Transcripts returned | Were transcripts returned to participants for comment and/or correction? | No | Not applicable |
| **Domain 3: analysis and ﬁndings** |  |  |  |
| *Data analysis* |  |  |  |
| 24. Number of data coders | How many data coders coded the data? | 2 data coders | Page 6 (Methods) |
| 25. Description of the coding tree | Did authors provide a description of the coding tree? | No | Not applicable |
| 26. Derivation of themes | Were themes identiﬁed in advance or derived from the data? | Derived from the data | Page 6 (Methods) |
| 27. Software | What software, if applicable, was used to manage the data? | NVivo12 | Page 6 (Methods) |
| 28. Participant checking | Did participants provide feedback on the ﬁndings? | No | Not applicable |
| *Reporting* |  |  |  |
| 29. Quotations presented | Were participant quotations presented to illustrate the themes/ﬁndings? Was each quotation identiﬁed? e.g. participant number | Yes | Pages 7-13 (Results) |
| 30. Data and ﬁndings consistent | Was there consistency between the data presented and the ﬁndings? | Yes | Pages 7-13 (Results) |
| 31. Clarity of major themes | Were major themes clearly presented in the ﬁndings? | Yes | Pages 7-13 (Results) |
| 32. Clarity of minor themes | Is there a description of diverse cases or discussion of minor themes? | Yes | Pages 7-13 (Results) |
