# Supplementary material for: “We’re sinking”: a qualitative interview-based study on stakeholder perceptions of structural and process limitations to the Canadian healthcare system
Source: Arch Public Health. 2024 Apr 25;82:56. doi: 10.1186/s13690-024-01279-4 (PMC11044548; doi:10.1186/s13690-024-01279-4)
Supplement: Supplementary file 2 — Supplementary Material 2 [file 13690_2024_1279_MOESM2_ESM.docx]

**Additional File 2: Interview Guide**


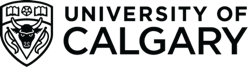


**Canadian Healthcare System Reform: Interview Guide**

**INTRODUCTION**

Thank you for agreeing to speak with us today. My name is [insert name] and I am a [study coordinator/research assistant] at [Dalhousie University/University of Calgary] working on this study. We appreciate you taking the time to meet with us today. This study aims to examine healthcare system reform in Canada. We are conducting interviews with public citizens, decision makers, healthcare leaders, academics and researchers across Canada.

This interview will focus on exploring your perceptions of the Canadian healthcare system and the changes that are needed to create a higher preforming system. This will include examining changes relevant for individual provinces and territories, and the contextual factors needed to facilitate change. These topics serve as a guide only. If there are other insights you would like to offer, we would like to hear them.

**Prior to this interview, we emailed you a link to the online informed consent form for you to sign electronically. We received your signed form. Thank you.**

**Do you have any questions?**

We want to remind you that participation in this interview is completely voluntary. There are no expected risks associated with participating in this study. Some people may find answering questions tiring. We will offer you breaks as needed. If at any point you feel uncomfortable with the interview and wish to end your participation you are free to do so. If you would like to skip a question or end the interview early, please let me know. There will be no direct benefit to you in participating in this study, but the information we gather will be used to understand current perceptions of Canada’s healthcare system and what changes could be made to help pursue effective reform. If you decide after the interview that you wish to withdraw your data, you will have one week following the interview. After one week, we cannot guarantee that we can remove your data as it will be combined with data from other interviews for analysis.

Before we start the interview, do you have any (other) questions? I also would like to remind you that we will be recording this discussion for transcription purposes.

<turn on recorder>

**INTERVIEW QUESTIONS**

**Topic 1: Current state of our health care system**

1. Considering the entirety of healthcare for all Canadians including and beyond your province/territory, how do you feel about the current Canadian healthcare system?
   1. What do you consider the best aspects/parts of the Canadian healthcare system?
2. How do you believe our healthcare system performs compared to other countries (e.g. UK, US, Sweden) which are similar in development and economic output?
   1. Probe: Performance indicators can include things such as health system outputs and outcomes.
   2. Probe: A common performance indicator of a nation’s health system is the infant mortality rate. The current infant mortality rate for Canada in 2022 is 4.055 deaths per 1000 live births, a 2.71% decline from 2021.
3. Just thinking “big picture,” what do you believe to be the biggest challenge facing the Canadian healthcare system?
   1. [If answer is pandemic focused] What about beyond the COVID-19 pandemic (e.g., consider post-pandemic)?
4. Could you describe any personal challenges that you or someone you know have experienced in the Canadian healthcare system? If any, how have these impacted your opinions on the current system? If none, have you learned about system challenges from others?
   1. Note: Make sure to query the direction (i.e., positive or negative) and the magnitude (i.e., large or small) of the impact of the challenges on their opinions of the healthcare system.

**Topic 2: Avenues of change**

1. In your opinion, what are the biggest changes needed to improve the Canadian healthcare system? If yes, why?

Probe: Phrased another way, if you could implement one change to the Canadian healthcare system tomorrow, what would it be and why?

- 1. Probe: Consider the differences in short-term vs long-term goals.

1. The next two questions will ask you about the components of the Canadian healthcare system that both facilitate and hinder reform or change in the healthcare system. The World Health Organization recognizes six components to a healthcare system. These include (1) service delivery, (2) health workforce, (3) health information systems, (4) medical products, vaccines and technologies, (5) health systems financing, (6) leadership and governance. [insert the WHO healthcare system components into Teams chat box].
   1. From your perspective, which system component has been most important for facilitating change within a Canadian context?
   2. On the flipside, which system component has been hindering change the most?
2. In your opinion, how well is Canada’s current healthcare system positioned to address future public health challenges?
   1. Some have argued that the current system is not sustainable for subpopulations, such as aging adults. What do you think we could do to improve the sustainability of the system for subpopulations?
   2. Which factors do you feel are most critical to creating a more sustainable healthcare system?
   3. What are the greatest barriers to creating this system?
3. As we know, healthcare is currently delivered through many different groups. For example, the federal government sends money to provinces/territories for their population’s healthcare. The provincial/territorial governments provide health insurance for their citizens. Both not for profit and profit groups deliver care (e.g., clinics hospitals).
   1. What are your thoughts on the current approach to providing healthcare?
      1. Probe: Consider the overarching systems approach to providing care.
   2. Do you believe the roles and responsibilities of certain groups should change? If yes, how so?

**Topic 3: Contextual factors influencing change**

1. Who do you believe are the actors responsible for promoting and/or implementing changes for improvement (e.g., governments, physicians, organizations, individuals)? What roles do the differing actors have?
2. How do you perceive contextual factors influencing potential healthcare system improvements? Contextual factors could include environmental factors (physical, social, attitudinal components), and personal factors (demographic characteristics)
   1. What role do you think political factors (like the federal party in power and the parties in power in provinces and territories) play in the potential for healthcare system change for improvement?

**CLOSING**

**Do you have any final thoughts?**

**Thank you for participating and sharing your opinions and experiences with me. If you have no further insights to chare, I will turn off the recorder now.**

<turn off recorder>

**DEMOGRAPHIC QUESTIONS**

We are collecting personal and family demographic information in order to describe our participants in aggregate. Contact information is only obtained to send participants an e-gift card. Please note that your demographic information and contact info will be stored in a password protected database that is only accessible to the study research team. If you are not comfortable answering any of the below questions you are welcome to skip any or all of those you do not wish to answer.

1. **What is your age, sex, gender?**
2. **Which ethnic, racial, or cultural group do you most closely self-identify with?**
3. **What is your highest level of education?**
4. **What is your employment status? (full-time/part-time/unemployed/retired)**
5. **Are you entitled to healthcare benefits with your employer?**
6. **What is your current professional title?**
7. **What is province do you currently live in?**

**As a thank you for you participation, we give our participants a $20 e-gift card. Do you have a preference between the following?**

- Chapters/Indigo
- Tim Horton
- Amazon

**What email address/mailing address do you wish to receive your e-gift card?**

**If participant would prefer another store, we can accommodate if reasonable.**
